# Tongue Microbiota Composition and Dental Caries Experience in Primary School Children

**DOI:** 10.1128/mSphere.01252-20

**Published:** 2021-04-28

**Authors:** Daixi Zhang, Toru Takeshita, Michiko Furuta, Shinya Kageyama, Mikari Asakawa, Koki Nambu, Yoshihisa Yamashita

**Affiliations:** a Section of Preventive and Public Health Dentistry, Division of Oral Health, Growth and Development, Faculty of Dental Science, Kyushu University, Fukuoka, Japan; b OBT Research Center, Faculty of Dental Science, Kyushu University, Fukuoka, Japan; University of Wisconsin-Madison

**Keywords:** 16S rRNA, children, dental caries, tongue microbiota

## Abstract

The tongue microbiota of elderly adults is composed of two cohabiting commensal groups and their ratios are related to the number of teeth with dental caries experience. In this study, the variation in the tongue microbiota of primary school children and its relationship with the dental caries experience were investigated. We examined the tongue microbiota of 138 children aged 6 to 7 years and 11 to 12 years (61 and 77 children, respectively) who underwent annual dental examinations. The bacterial composition was determined by sequencing the V1-V2 region of the 16S rRNA gene. Cooccurrence network analysis indicated two groups of cohabiting predominant commensals in the tongue microbiota of children. The microbiota in children without a history of dental caries showed significantly higher relative abundances of one of the cohabiting groups, primarily composed of Neisseria subflava, Porphyromonas pasteri, and Fusobacterium periodonticum, compared to that in children with a history of dental caries, which is consistent with that of elderly adults with fewer teeth with dental caries experience. Linear discriminant analysis effect size (LEfSe) further identified Streptococcus oralis subsp. *dentisani*, belonging to the aforementioned commensal group, as a discriminant species in children without dental caries experience aged 6 to 7 years and 11 to 12 years. Our results describe the tongue microbiota composition of primary school children without history of dental caries and support the possibility that dental caries experience is accompanied by a shift in the tongue microbiota.

**IMPORTANCE** Dental caries is now considered to be caused by acids produced by the overall dental plaque microbiota rather than by specific pathogens. This study focused on the relationship between dental caries experience and the variations in tongue microbiota, which is adjacent but separate from the dental plaque microbiota. Our results demonstrated that the tongue microbiota of primary school children with no history of dental caries experience was composed of predominant commensals with different relative abundances compared to those present in children with dental caries experience, suggesting that dental caries experience is accompanied by a shift in the tongue microbiota. The maintenance of a healthy tongue microbiota may indirectly contribute to the prevention of dental caries.

## Abstract (precis)

Dental caries is now considered to be caused by acids produced by the overall dental plaque microbiota rather than by specific pathogens. This study focused on the relationship between dental caries experience and the variations in tongue microbiota, which is adjacent but separate from the dental plaque microbiota.

## INTRODUCTION

Dental caries is a common chronic oral disease characterized by the demineralization of tooth tissue and decomposition of organic matter, which leads to cavitation or tooth decay (1). The etiology of this disease involves acid production by the dental plaque microbiota, which accumulates on the tooth surface. Of the bacterial members, mutans streptococci were implicated as the single causal agent of the disease in earlier studies, and their virulence has been studied extensively (2, 3). However, even though the microorganism is undoubtedly associated with the initiation of dental caries (2, 4), its presence is not indispensable for dental caries development. Currently, the prevailing “ecological hypothesis” proposes that changes in the oral environment alter the dental plaque microbiota into an aciduric one, composed of diverse bacteria with acidogenic potential, which leads to the development and progression of dental caries (5, 6).

The tongue microbiota is another complex oral microbial community that is adjacent but unattached to the plaque microbiota, and has a community composition distinct from that of the dental plaque microbiota (7–9). Interestingly, a population-based study in elderly adults showed a significant relationship between the ratio of two groups of cohabiting predominant commensals in the tongue microbiota and the number of teeth with dental caries experience (10). A similar result was observed in another population-based study in middle-aged and elderly adults analyzing salivary microbial populations (11), which showed dominant tongue microbes (7–9). The findings from these studies indicate that dental caries development might be accompanied by dysbiotic microbiota on decayed teeth as well as elsewhere in the oral cavity. However, there remains a possibility that the shift in the tongue microbiota in middle-aged and elderly adults is caused by other changes occurring during their life course, including periodontal diseases and tooth loss.

Here, we studied the tongue microbiota of primary school children aged 6 to 7 years and 11 to 12 years with recent complaints of dental caries. We determined the tongue microbiota composition and analyzed the variation in the composition and its relationship with dental caries.

## RESULTS

In this study, the tongue microbiota composition of 138 primary school children aged 6 to 7 years and 11 to 12 years (61 and 77 children, respectively) was studied based on the sequencing of the 16S rRNA gene using a next-generation sequencer, the Ion PGM system (Thermo Fisher Scientific, Waltham, MA, USA). The samples provided 3,478,851 quality reads corresponding to the V1-V2 regions of the 16S rRNA gene sequences, which were subsequently assigned to 302 species-level operational taxonomic units (OTUs) based on 97% sequence similarity. Of these, 21 OTUs corresponding to typical oral commensals were the predominant taxa present in the tongue microbiota of the children (Table 1), and their mean relative abundance in each individual was 79.0 ± 6.4% (mean ± standard deviation [SD]).

**TABLE 1:** Twenty-one predominant operational taxonomic units (OTUs) with mean relative abundances of >1% in the tongue microbiota of 138 primary school children

OTU no.	Bacterial taxa corresponding to each OTU^*a*^	% Mean relative abundance ± SD	% Detection rate
OTU4	Neisseria subflava (476)	12.1 ± 9.2	100
OTU3	Prevotella melaninogenica (469)	12.0 ± 7.3	100
OTU7	Streptococcus salivarius (755)	7.4 ± 6.0	100
OTU10	Veillonella parvula (161)	6.8 ± 3.6	100
OTU2	Rothia mucilaginosa (681)	5.1 ± 4.0	99.3
OTU1	Fusobacterium periodonticum (201)	4.5 ± 3.5	100
OTU6	Haemophilus parainfluenzae (718)	4.4 ± 4.5	100
OTU9	Granulicatella adiacens (534)	3.9 ± 2.1	100
OTU8	Porphyromonas pasteri (279)	3.8 ± 4.0	97.1
OTU5	Prevotella histicola (298)	3.5 ± 4.5	87.7
OTU30	Streptococcus infantis (431)	2.2 ± 2.1	100
OTU25	Actinomyces graevenitzii (866)	2.2 ± 2.9	94.9
OTU13	Prevotella pallens (714)	1.6 ± 1.3	94.9
OTU11	*Alloprevotella* sp. (308)	1.5 ± 1.8	95.7
OTU22	Actinomyces odontolyticus (701)	1.3 ± 0.9	99.3
OTU19	Genus *Streptococcus*^*b*^	1.3 ± 1.5	98.6
OTU170	Rothia mucilaginosa (681)	1.1 ± 0.9	99.3
OTU15	*Leptotrichia* sp. (417)	1.1 ± 2.3	86.2
OTU46	*Streptococcus* sp. (064)	1.1 ± 0.8	100
OTU12	Solobacterium moorei (678)	1.1 ± 0.9	97.8
OTU133	*Prevotella* sp. (313)	1.0 ± 1.7	79.0

a Oral taxon IDs in eHOMD are given in parentheses following bacterial names.

b No blast hit with ≥98.5% identity was found in the expanded Human Oral Microbiome database (eHOMD).

**FIG 1:**
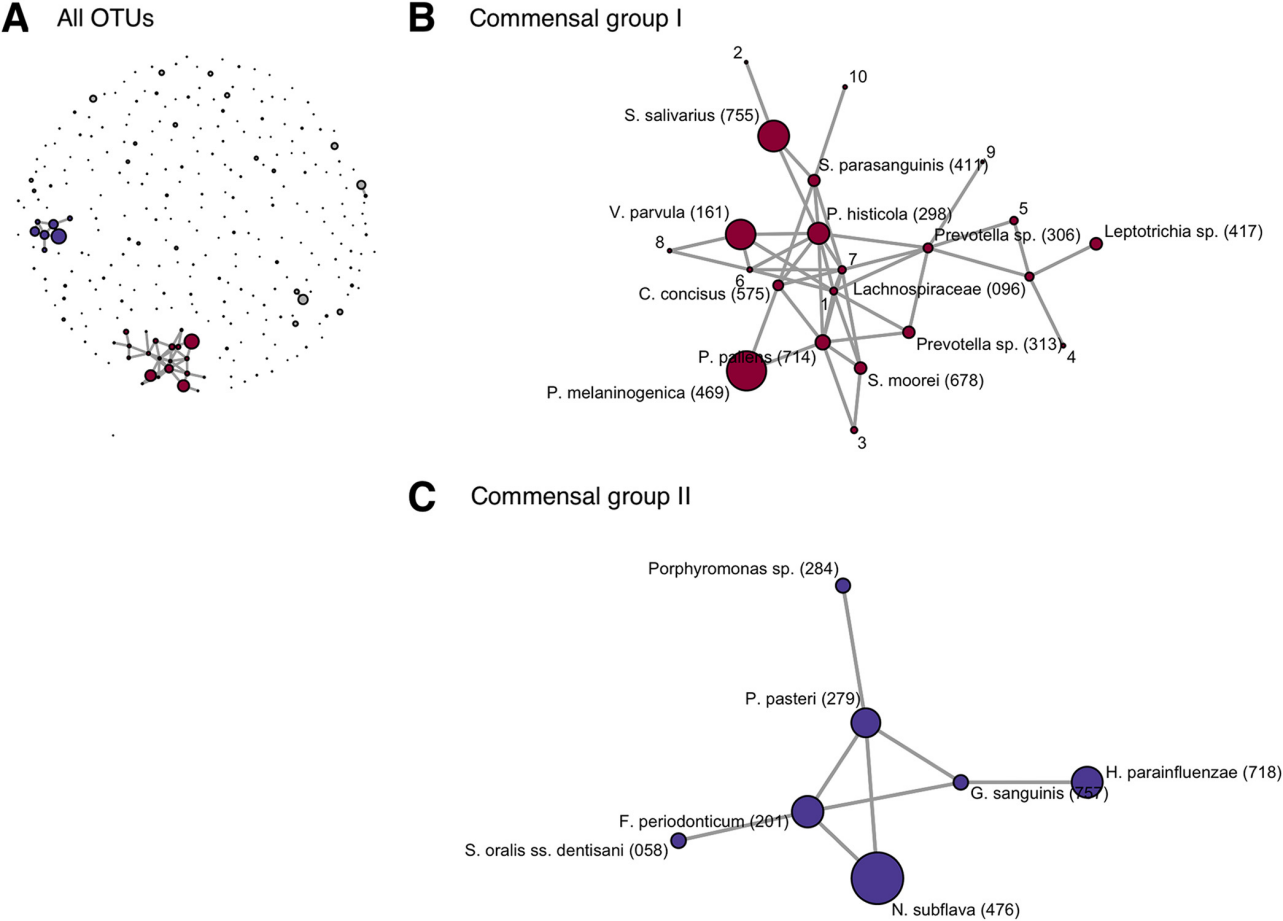
Cooccurrence network in the tongue microbiota of 138 children built from SparCC correlation coefficients between sequence abundances. Each node corresponds to a distinct operational taxonomic unit (OTU) and correlations with values greater than 0.5 and *P* values less than 0.001 are represented as edges. The size of each node indicates the mean relative abundance of each OTU. (A) All OTUs are shown as nodes in the diagram. The OTUs belonging to two major networks (commensal group I and II) are colored red and blue, respectively. (B) Commensal group I. The bacterial name corresponding to each OTU is described at each node. Oral taxon IDs from the expanded Human Oral Microbiome database are provided in parentheses following the bacterial name. The bacterial names corresponding to minor OTUs (relative abundance < 0.5%) are indicated using numbers and described in Table S1 in the supplemental material. (C) Commensal group II. The bacterial name corresponding to each OTU is described at each node. Oral taxon IDs in the expanded Human Oral Microbiome database are provided in parentheses following the bacterial name.

Cooccurrence network analysis using FastSpar (12) suggested that the predominant commensals were a part of two distinct cohabiting groups in the tongue microbiota (Fig. 1A). The members of each cohabiting group are listed in Table S1 in the supplemental material. The first group was composed of bacterial taxa such as Prevotella histicola, Veillonella parvula, Streptococcus salivarius, Prevotella pallens, and Prevotella melaninogenica (commensal group I, Fig. 1B), whereas the second group was composed of Neisseria subflava, Porphyromonas pasteri, Fusobacterium periodonticum, Haemophilus parainfluenzae, and S. oralis subsp. *dentisani* (commensal group II, Fig. 1C). There was an inverse relationship between the relative abundances of the two commensal groups (Spearman’s correlation coefficient = −0.91) (Fig. S1).

10.1128/mSphere.01252-20.1 FIG S1 Relative abundances of the two cohabiting commensal groups in the tongue microbiota. Download FIG S1, PDF file, 0.2 MB. Copyright © 2021 Zhang et al. 2021 Zhang et al. https://creativecommons.org/licenses/by/4.0/ This content is distributed under the terms of the Creative Commons Attribution 4.0 International license.

10.1128/mSphere.01252-20.6 TABLE S1 Bacterial taxa corresponding to OTUs belonging to commensal groups I and II. Download Table S1, DOCX file, 0.02 MB. Copyright © 2021 Zhang et al. 2021 Zhang et al. https://creativecommons.org/licenses/by/4.0/ This content is distributed under the terms of the Creative Commons Attribution 4.0 International license.

**FIG 2:**
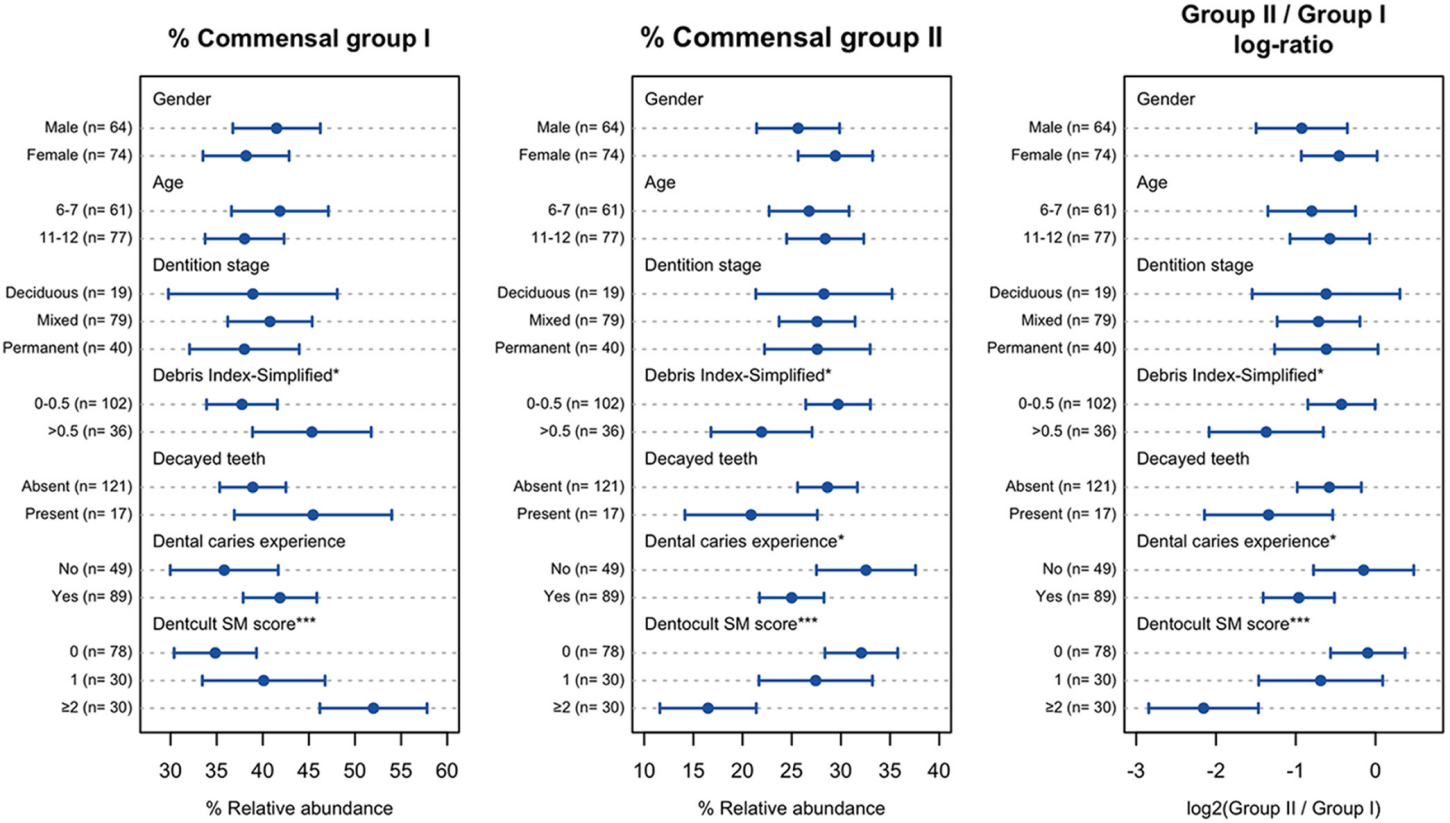
Relative abundances of two cohabiting commensal groups in the tongue microbiota and the ratios of group II commensals to group I commensals (log 2-transformed) of primary school children with different conditions. The dots indicate the mean, and the error bars indicate the 95% confidence interval (CI). ***, *P < *0.001; *, *P < *0.05 by Student’s *t* test (two categories) or analysis of variance (three categories).

The relative abundances of the commensal groups in the tongue microbiota varied according to the dental health conditions, whereas no notable differences were observed between children of different genders, ages, and dentition stages (Fig. 2). The commensal group II was significantly more predominant in children without dental caries experience and those with better dental hygiene (lower debris index-simplified [DI-S]) (13). A higher relative abundance of this commensal group was also observed in the microbiota of children with lower mutans streptococci levels in their saliva (lower Dentocult SM score). Alternatively, commensal group I was more predominant in the microbiota of children with higher Dentocult SM and DI-S scores. The ratios of group II commensals to group I commensals were significantly higher in children without dental caries experience and those with lower Dentocult SM and DI-S scores.

**FIG 3:**
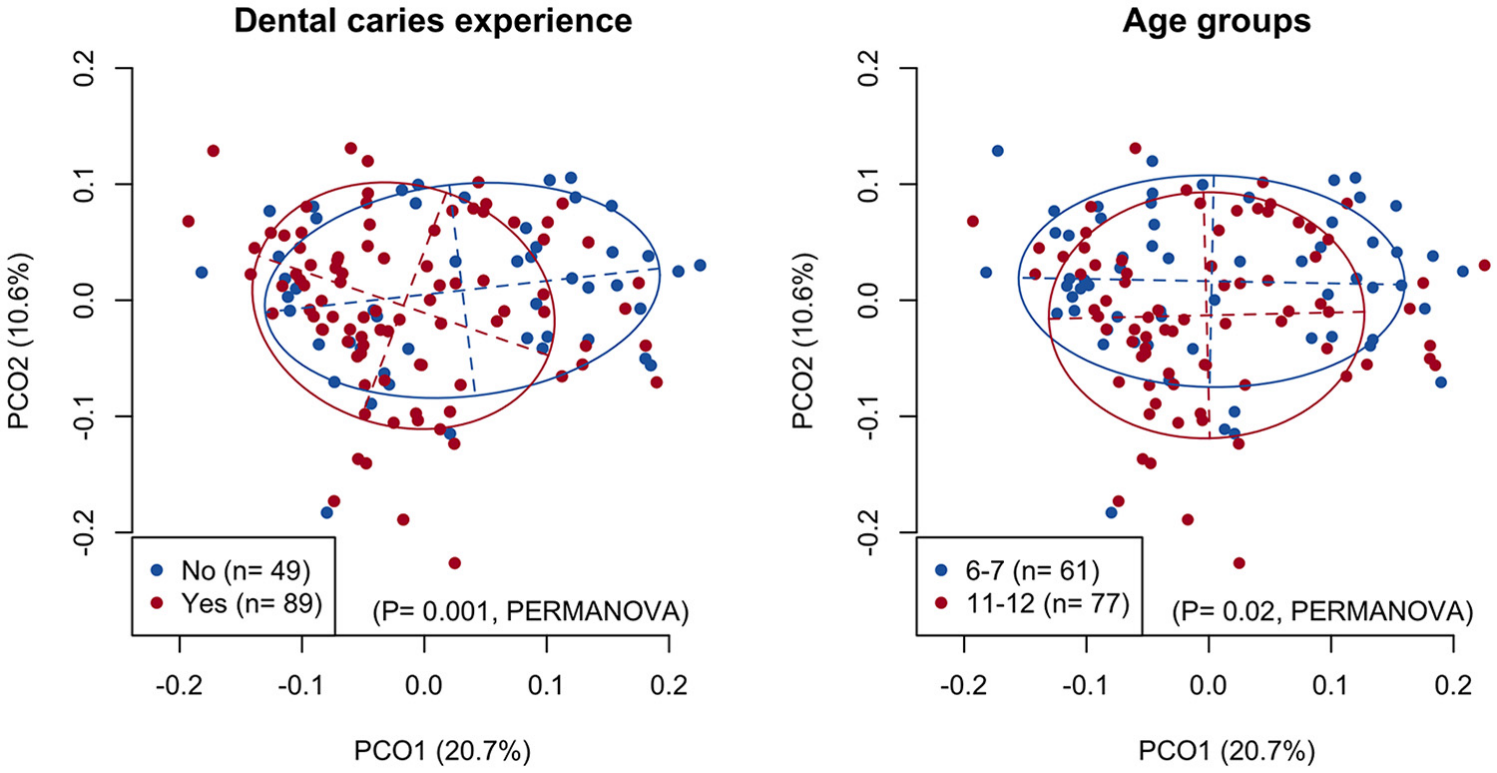
A principal coordinate analysis plot showing the similarity relationship among the tongue microbiota of 138 primary school children using an unweighted UniFrac distance metric. The points corresponding to the children with different categories of dental caries experience (left panel) and age groups (right panel) are depicted using different colors. The two axes explain 20.7% and 10.6% of the variance, respectively.

No notable relationship was observed between these clinical parameters and the alpha diversity indices of the tongue microbiota, except for a higher microbial richness observed in children with a higher DI-S score (Fig. S2). In contrast, the principal-coordinate analysis (PCoA) plot based on unweighted UniFrac metrics showed differences between children with and without dental caries experience in the direction of principal coordinate 1 and differences between children aged 6 to 7 years and 11 to 12 years in the direction of principal coordinate 2 (Fig. 3). The significant relationship with the overall composition of the tongue microbiota was confirmed by permutational multivariate analysis of variance (PERMANOVA *F* = 2.92 and *P* = 0.001; *F* = 2.04 and *P* = 0.02, respectively), whereas there was no significant relationship with gender, dentition stage, DI-S, or a presence of decayed teeth. Significant differences were observed in the relative abundances of *Streptococcus* and *Neisseria* among the four groups categorized by age and dental caries experience (Fig. S3). The children could also be differentiated according to the mutans streptococci levels in the saliva in the direction of principal coordinate 1 in the UniFrac plot (PERMANOVA *F* = 2.72 and *P* < 0.001) (Fig. S4).

10.1128/mSphere.01252-20.2 FIG S2 Alpha diversity indices (number of identified operational taxonomic units [OTUs] for microbial richness and Shannon diversity index for microbial diversity) in the tongue microbiota of primary school children with different conditions. The dots indicate the mean, and the error bars indicate the 95% CI. *P < 0.05 using Student’s *t* test (two categories) or analysis of variance (three categories). Download FIG S2, PDF file, 0.2 MB. Copyright © 2021 Zhang et al. 2021 Zhang et al. https://creativecommons.org/licenses/by/4.0/ This content is distributed under the terms of the Creative Commons Attribution 4.0 International license.

10.1128/mSphere.01252-20.3 FIG S3 Relative abundances of predominant bacterial genera (mean relative abundances of >2%) in the tongue microbiota of children. The children were classified based on their age groups (6 to 7 and 11 to 12 years) and dental caries experience (CE, caries-experienced; CF, caries-free). *, *P* < 0.05 using analysis of variance. Download FIG S3, PDF file, 0.2 MB. Copyright © 2021 Zhang et al. 2021 Zhang et al. https://creativecommons.org/licenses/by/4.0/ This content is distributed under the terms of the Creative Commons Attribution 4.0 International license.

10.1128/mSphere.01252-20.4 FIG S4 A principal coordinate analysis plot showing the similarity relationship among the tongue microbiota from 138 primary school children using an unweighted UniFrac distance metric. The points corresponding to the children with different levels of mutans streptococci in saliva are depicted in different colors. The two axes explain 20.7% and 10.6% of the variance, respectively. Download FIG S4, PDF file, 0.3 MB. Copyright © 2021 Zhang et al. 2021 Zhang et al. https://creativecommons.org/licenses/by/4.0/ This content is distributed under the terms of the Creative Commons Attribution 4.0 International license.

**FIG 4:**
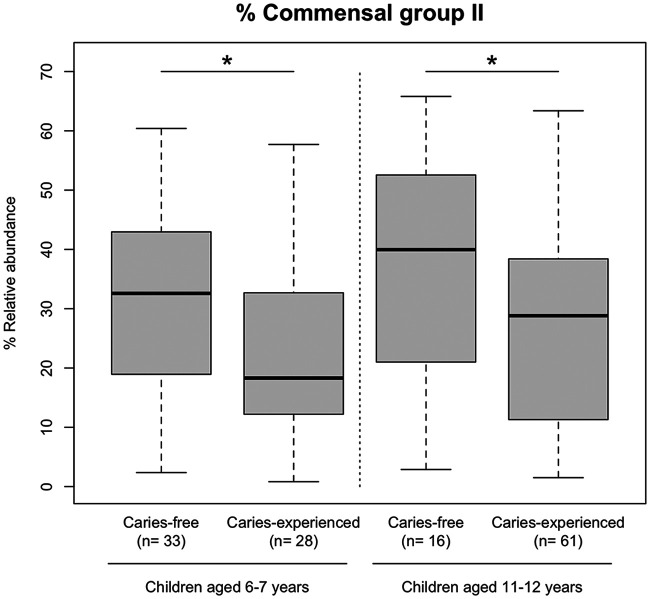
Relative abundances of commensal group II in the tongue microbiota of primary school children aged 6 to 7 years and 11 to 12 years with and without dental caries experience; *, *P < *0.05, Student’s *t* test.

While the overall tongue microbiota composition was different between children aged 6 to 7 years and 11 to 12 years, the commensal group II was significantly more predominant in caries-free children than in children with dental caries experience from either age group (6 to 7 years and 11 to 12 years) (Fig. 4). Linear discriminant analysis effect size (LEfSe) further revealed ten and six taxa associated with the presence or absence of dental caries experience in children aged 6 to 7 years and 11 to 12 years, respectively (Tables 2 and 3). Of these, S. oralis subsp. *dentisani*, which belongs to commensal group II, was identified as a discriminant taxon associated with the absence of dental caries experience in both age groups. In contrast, Streptococcus parasanguinis, a member of the commensal group I, was detected in the tongue microbiota of children with dental caries experience in both age groups.

**TABLE 2:** Bacterial taxa corresponding to differentially abundant OTUs in the tongue microbiota of children aged 6 to 7 years with and without dental caries experience

OTU no.	Bacterial taxa corresponding to each OTU^*a*^	% Relative abundance ± SD		LDA score^*c*^
		Without previous dental caries (*n* = 33)	With previous dental caries (*n* = 28)	
Differentially abundant in children with dental caries experience				
OTU10	Veillonella parvula (161)	6.30 ± 3.15	8.53 ± 4.12	4.09
OTU38	Streptococcus parasanguinis (411)	0.66 ± 0.76	1.44 ± 1.66	3.62
OTU151	Genus *Streptococcus*^*b*^	0.1 ± 0.16	0.34 ± 0.43	3.13
OTU136	*Leptotrichia* sp. (498)	0 ± 0	0.02 ± 0.05	3.07
OTU188	Genus *Prevotella*^*b*^	0 ± 0	0.01 ± 0.02	3.04
OTU168	Genus *Fusobacterium*^*b*^	0 ± 0	0.01 ± 0.01	3.02
Differentially abundant in caries-free children				
OTU103	Genus *Fusobacterium*^*b*^	0.01 ± 0.01	0.00 ± 0.01	3.10
OTU66	Streptococcus australis (073)	0.58 ± 0.73	0.39 ± 0.68	3.13
OTU129	Order *Clostridiales*^*b*^	0.01 ± 0.01	0 ± 0	3.48
OTU34	Streptococcus oralis subsp. *dentisani* (058)	1.49 ± 1.47	0.55 ± 0.87	3.71

a Oral taxon IDs in eHOMD are given in parentheses following bacterial names.

b No blast hit with ≥98.5% identity was found in the expanded Human Oral Microbiome database (eHOMD).

c Linear discriminant analysis (LDA) effect size (LEfSe) was conducted, and OTUs with a high LDA score (>3.0) are shown.

**TABLE 3:** Bacterial taxa corresponding to differentially abundant OTUs in the tongue microbiota of children aged 11 to 12 years with and without dental caries experience

OTU no.	Bacterial taxa corresponding to each OTU^*a*^	% Relative abundance ± SD		LDA score^*b*^
		Without previous dental caries (*n* = 16)	With previous dental caries (*n* = 61)	
Differentially abundant in children with dental caries experience				
OTU5	Prevotella histicola (298)	1.92 ± 2.81	3.93 ± 5.09	4.03
OTU25	Actinomyces graevenitzii (866)	1.33 ± 2.11	2.36 ± 3.33	3.74
OTU26	*Lachnospiraceae* bacterium (096)	0.08 ± 0.23	0.62 ± 1.51	3.42
OTU38	Streptococcus parasanguinis (411)	0.67 ± 0.78	1.05 ± 0.86	3.40
Differentially abundant in caries-free children				
OTU161	Actinomyces johnsonii (849)	0.00 ± 0.01	0 ± 0	3.45
OTU34	Streptococcus oralis subsp. *dentisani* (058)	1.80 ± 1.73	0.7 ± 0.86	3.70

a Oral taxon IDs in eHOMD are given in parentheses following bacterial names.

b Linear discriminant analysis (LDA) effect size (LEfSe) was conducted, and OTUs with a high LDA score (>3.0) are shown.

## DISCUSSION

This study demonstrated that the tongue microbiota of primary school children is dominated by common oral commensals, including *N. subflava* and *P. melaninogenica* (Table 1), and is composed of two competing cohabiting groups (Fig. 1). The two cohabiting groups were mostly consistent with those found in the tongue microbiota of elderly adults in a previous study (10); the findings suggest that the tongue microbiota, composed of specific indigenous taxa, has already been established in childhood during the transitional stage between primary and permanent dentition.

This study also characterized the tongue microbiota of primary school children with no history of dental caries. One of the two cohabiting predominant commensal groups, primarily composed of *N. subflava*, *H. parainfluenzae*, *P. pasteri*, and F. periodonticum (commensal group II), was significantly more predominant in the microbiota of children without dental caries experience (Fig. 2), which is consistent with the microbiota composition of elderly adults with less dental caries experience (10). Significant differences in the relative abundances of the commensal group were observed in the microbiota of children aged 6 to 7 years and 11 to 12 years who were affected by dental caries more recently (Fig. 2, Fig. 4). These results support the possibility that dental caries experience is accompanied by a shift in the tongue microbiota.

The genera *Neisseria*, *Haemophilus*, and *Rothia* were associated with good oral health, whereas *Prevotella* species (predominant in commensal group I) and Streptococcus mutans were implicated in dental caries in a recent study using a shotgun metagenomics approach (14). Another metagenomic study demonstrated that the genera *Prevotella*, *Veillonella*, and *Atopobium* (belonging to commensal group I in this study) were more abundant in the supragingival microbiota of children with dental caries than in those without dental caries (15). In addition, considering that oral *Prevotella* species have been implicated as dental caries-associated taxa in several studies previously (16–19), our results are consistent with previous evidence. However, a large-scale twin study showed that Prevotella pallens and *Veillonella* spp. are heritable taxa associated with the absence of dental caries, which is discordant with our findings (20). Further careful investigation is needed to elucidate the relationship between the ratios of the abundances of predominant oral commensals and dental caries.

This cross-sectional study was unable to clarify whether the shifted tongue microbiota is responsible for dental caries or is a result of the development of dental caries. Nevertheless, considering that salivary levels of mutans streptococci were clearly correlated with the ratio of the two cohabiting commensals in the tongue microbiota (Fig. 2), it is unlikely that dental restorations, such as resin and metal, result in a shift in the tongue microbiota. The shift in the tongue microbiota coincides with the overgrowth of mutans streptococci in the plaque microbiota induced by an oral environment susceptible to dental caries. Future longitudinal and microbiological studies will help clarify the relationship between the tongue microbiota and susceptibility to dental caries.

Of the members of the commensal group II, S. oralis subsp. *dentisani* was identified as a key discriminant species for children with no history of dental caries experience from both age groups (6 to 7 years and 11 to 12 years) (Tables 2 and 3). S. oralis subsp. *dentisani* was first isolated from the dental plaque of caries-free individuals (21). This species was further reported to inhibit the growth of oral pathogens, including mutans streptococci, through the production of potent bacteriocins, as well as through its pH-buffering capacity via an arginolytic pathway (22), which makes it promising as an oral probiotic with anti-caries potential (23). Our results further support the potential of this taxon as a species beneficial for oral health, although its role in lowering susceptibility to dental caries remains unclear.

Another noteworthy species in this study was *S. parasanguinis*, which was a discriminant taxon for children with dental caries experience from either age group (Tables 2 and 3) and was previously implicated in the dental plaque microbiota of children with severe early childhood dental caries (18). However, these commensal streptococci were also reported to restrict the growth and biofilm formation of S. mutans in the presence of nitrite (24). Therefore, careful attention should be paid to the association of *S. parasanguinis* with dental caries.

This study assessed the tongue microbiota composition in primary school children with no previous experience of dental caries based on the results of annual dental examination throughout their school years. However, decayed deciduous teeth lost prior to the first dental examination were not accounted for. Nevertheless, all children were younger than 7 years and 3 months in age at their first dental examination and had almost complete primary dentition except for the occasional loss of some incisors. The details of dental caries experience, including that in deciduous teeth, were mostly covered in this study. In contrast, the absence of dental caries diagnosis based on the observation of enamel caries is a major limitation of this study. Dental caries is evaluated based on visual inspection during dental examination conducted in schools; therefore, only obvious tooth decay without air drying is considered in dental caries diagnosis. A more rigorous diagnosis, such as that based on the International Caries Detection and Assessment System (ICDAS) criteria (25), would be required for the accurate evaluation of dental caries experience. Another limitation is this study was the inability to prevent the children from cleaning their tongues before sample collection. Although the tongue microbiota composition is relatively stable over time (26), it is possible that the tongue microbiota of some children differs from the usual composition.

Our results suggest that a shifted tongue microbiota is present in the background of dental caries. Although dental caries is undoubtedly caused by dysbiosis in the dental plaque microbiota, a broader view of the entire oral cavity as a microbial consortium might add a fresh perspective in defining the etiology of dental caries.

## MATERIALS AND METHODS

### Ethics approval.

All parents or guardians of child participants understood the nature of the study and provided written informed consent. The Ethics Committee of Kyushu University approved the study design, as well as the procedure for obtaining informed consent (reference no. 30-303). All experiments were performed in accordance with the approved guidelines.

### Study population, sample collection, and dental examination.

We enrolled 173 children aged 6 to 7 years and 11 to 12 years in two primary schools in Hisayama town, Fukuoka prefecture, Japan, who underwent annual dental examinations. Prior to the dental examination, the salivary levels of mutans streptococci were evaluated using a Dentocult SM strip (Orion Diagnostica, Espoo, Finland), and a sample of the tongue coating was collected by swabbing the center of the tongue dorsum five times using a HydraFlock swab (Puritan Medical Products, Guilford, ME, USA). The collected tongue coating samples were stored in 200 μl of lysis buffer containing 10 mM Tris-HCl, 1 mM EDTA, and 1% sodium dodecyl sulfate and transferred to the laboratory on ice. In addition to determining the number of present, decayed, or filled teeth, the oral hygiene status was evaluated using the DI-S score of the simplified oral hygiene index (OHI-S) (13) for six selected teeth (positions 16, 11, 26, 36, 41, and 46) based on visual inspection during the dental examination. The target teeth were modified to six deciduous teeth (positions 55, 51, 65, 75, 81, and 85) in children aged 6 to 7 years because they had primary dentition. For children aged 6 to 7 years, the absence of decayed or filled deciduous and permanent teeth at this dental examination was considered the absence of dental caries experience. However, for children aged 11 to 12 years, the absence of dental caries experience was defined based on the continued absence of decayed and filled teeth in the dental examination records throughout their school years, including the record for the current year. Three children without decayed and filled teeth in this examination were excluded from the analysis owing to incomplete dental examination data recorded during their school years. The number of decayed and filled deciduous teeth in children aged 6 to 7 years and 11 to 12 years ranged from 0 to 10 (median, 0) and 0 to 6 (median, 0), respectively. The number of decayed and filled permanent teeth in children aged 6 to 7 years and 11 to 12 years ranged from 0 to 3 (median, 0) and 0 to 3 (median, 0), respectively.

### Bacterial community analysis based on 16S rRNA gene sequencing.

DNA was extracted from the tongue coating samples using the bead-beating method, as described previously (27). Thirty children were excluded from the analysis as insufficient DNA was extracted due to a technical failure in the process. The V1-V2 region of the 16S rRNA gene from each sample was amplified using the following primers: 8F (5′-AGA GTT TGA TYM TGG CTC AG-3′) with the Ion Torrent adaptor A and a sample-specific 8-base tag sequence, and 338R (5′-TGC TGC CTC CCG TAG GAG T-3′) with the Ion Torrent trP1 adaptor sequence. The V1-V2 region can be used to discriminate among most *Streptococcus* species, which is the most predominant and clinically important oral taxon at the species level, and its use is currently recommended in studies on the oral microbiota (28). PCR amplification, purification, and quantification were performed as described previously (11). The indexed PCR amplicons were normalized and pooled to prepare a sequencing library. Emulsion PCR and library enrichment were performed using the Ion One Touch 2 system (Thermo Fisher Scientific) with an Ion PGM Hi-Q View OT2 kit (Thermo Fisher Scientific). Sequencing was conducted using an Ion PGM system (Thermo Fisher Scientific) with an Ion PGM Hi-Q View sequencing kit (Thermo Fisher Scientific).

### Data processing.

The obtained sequence reads were filtered based on their quality using a script written in R. The reads were excluded from the analysis if they contained ≤240 bases, had an average quality score of ≤25, did not include the correct forward primer sequence, did not include the correct reverse primer sequence (one mismatch was allowed), or had >7 nucleotide homopolymer. The remaining reads were assigned to the appropriate sample by examining the tag sequences. Operational taxonomic unit (OTU) clustering was conducted using UPARSE (29), with a minimum pairwise identity of 97%. The taxonomy of representative sequences of each OTU was determined using BLAST against 998 16S rRNA gene sequences (eHOMD 16S rRNA RefSeq, version 15.1) deposited in the expanded Human Oral Microbiome Database (30). Nearest-neighbor species with 98.5% identity were identified as candidate taxa for each OTU. The taxonomy of sequences with no appropriate hits was determined using the RDP classifier (31) with a minimum support threshold of 80% up to the genus level. The alpha diversity index and the relative abundance of each OTU were calculated following rarefaction with a depth of 5,000 reads per sample using R. The rarefaction curves are shown in Fig. S5. Two children were excluded from the analysis as the number of obtained reads was insufficient.

10.1128/mSphere.01252-20.5 FIG S5 Rarefaction curve for a number of OTUs per samples. Download FIG S5, PDF file, 0.4 MB. Copyright © 2021 Zhang et al. 2021 Zhang et al. https://creativecommons.org/licenses/by/4.0/ This content is distributed under the terms of the Creative Commons Attribution 4.0 International license.

### Statistical analysis.

All statistical analyses were conducted using R version 3.5.2. Cooccurrence network analysis was performed for 283 OTUs detected from ≥2 individuals using FastSpar (12), which is a fast and parallelizable implementation of the SparCC algorithm (32), with 5,000 bootstraps. Cooccurrence relationships with correlation values of >0.5 and *P* values of <0.001 were represented as edges, and displayed using the gplot function in the sna library. The relative abundances of each bacterial group were compared using Student’s *t* test between two categories and analysis of variance (ANOVA) among three or more categories. The similarity relationship of overall tongue microbiota structure, assessed using the UniFrac metric (33), was presented in a principal-coordinate analysis (PCoA) plot. Permutational multivariate analysis of variance (PERMANOVA) was used to assess the effects of general and dental conditions on tongue microbiota composition based on 9,999 permutations. Discriminant OTUs of children with or without dental caries experience were explored using the LEfSe approach (34).

### Data availability.

The obtained sequence data were deposited in the DDBJ Sequence Read Archive under accession no. DRA011686.
