# Supplementary figures and images for: Tongue Microbiota Composition and Dental Caries Experience in Primary School Children
Source: mSphere. 2021 Apr 28;6(2):e01252-20. doi: 10.1128/mSphere.01252-20 (PMC8092142; doi:10.1128/mSphere.01252-20)

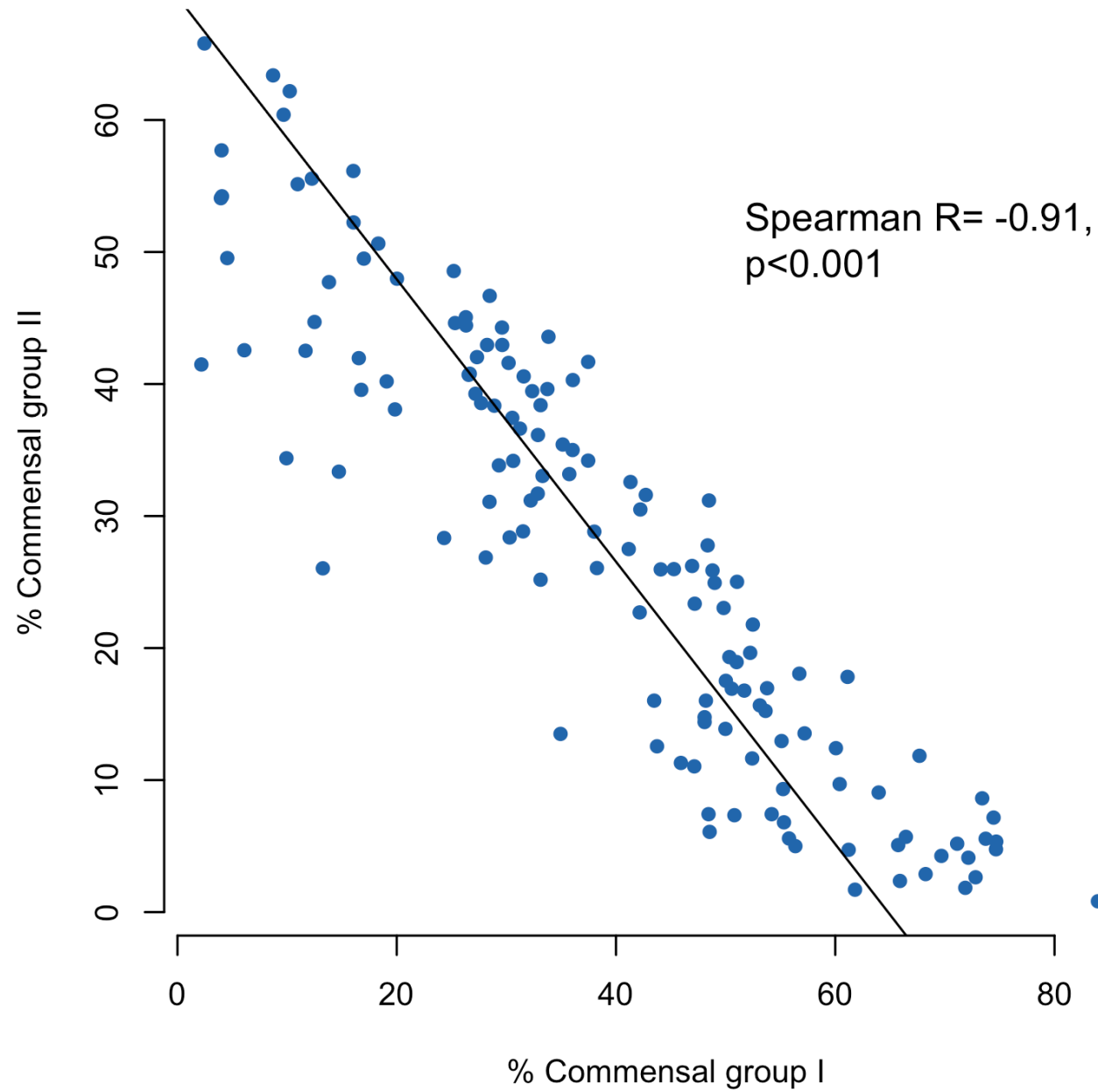

Supplement: FIG S1 [file mSphere.01252-20-sf001.pdf]

## No. of identified OTUs

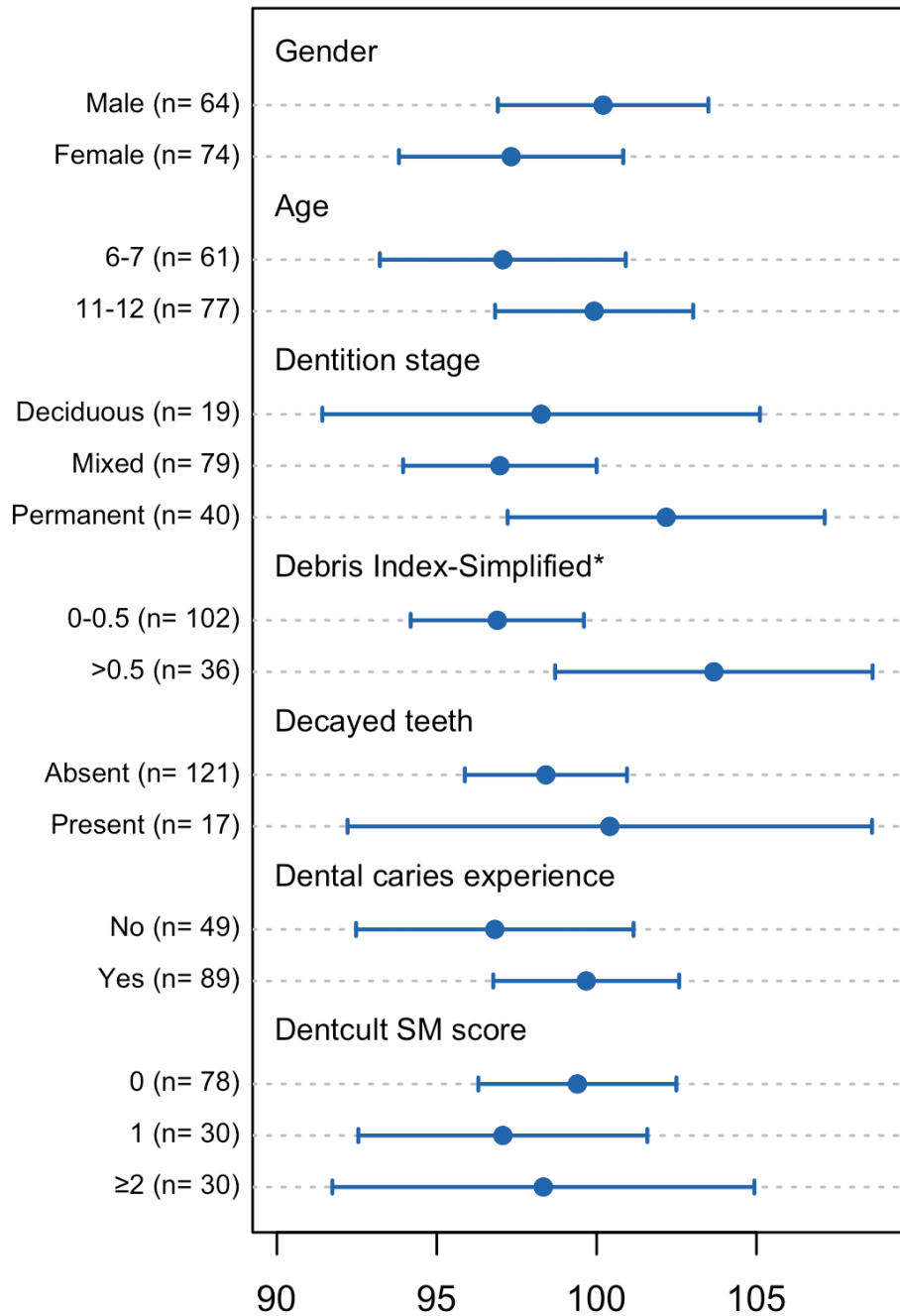

## Shannon diversity index

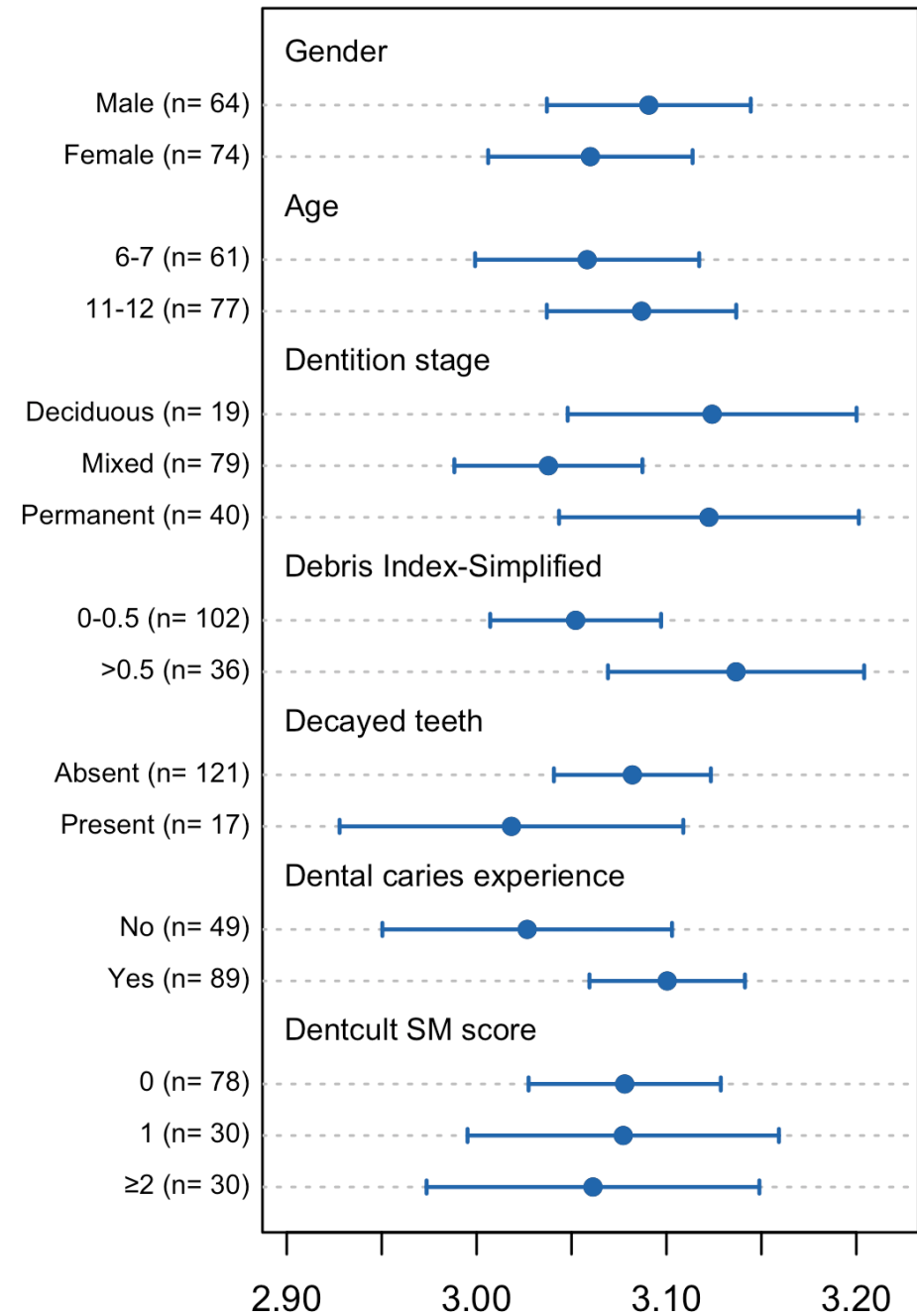

Supplement: FIG S2 [file mSphere.01252-20-sf002.pdf]

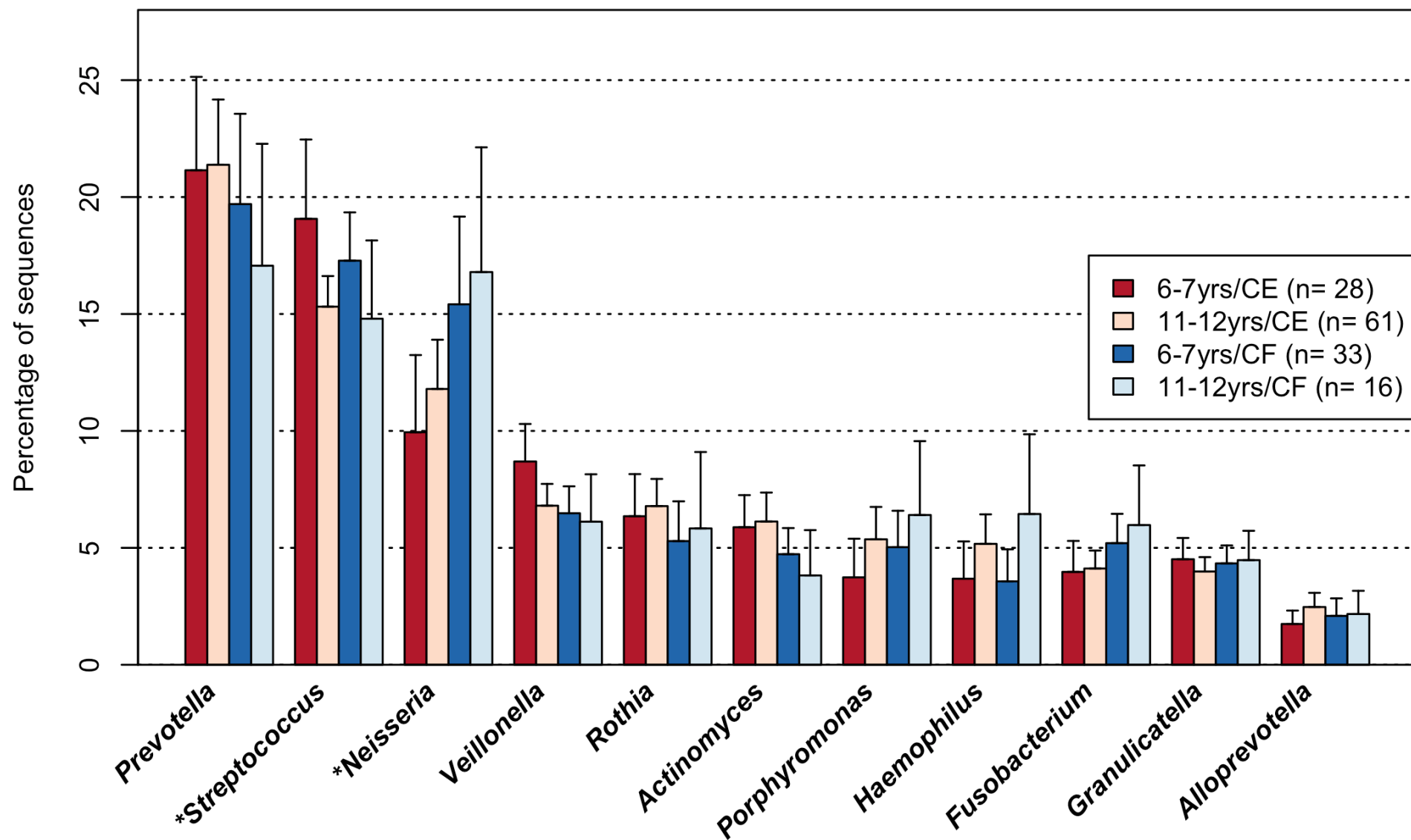

Supplement: FIG S3 [file mSphere.01252-20-sf003.pdf]

# Dentocult SM score

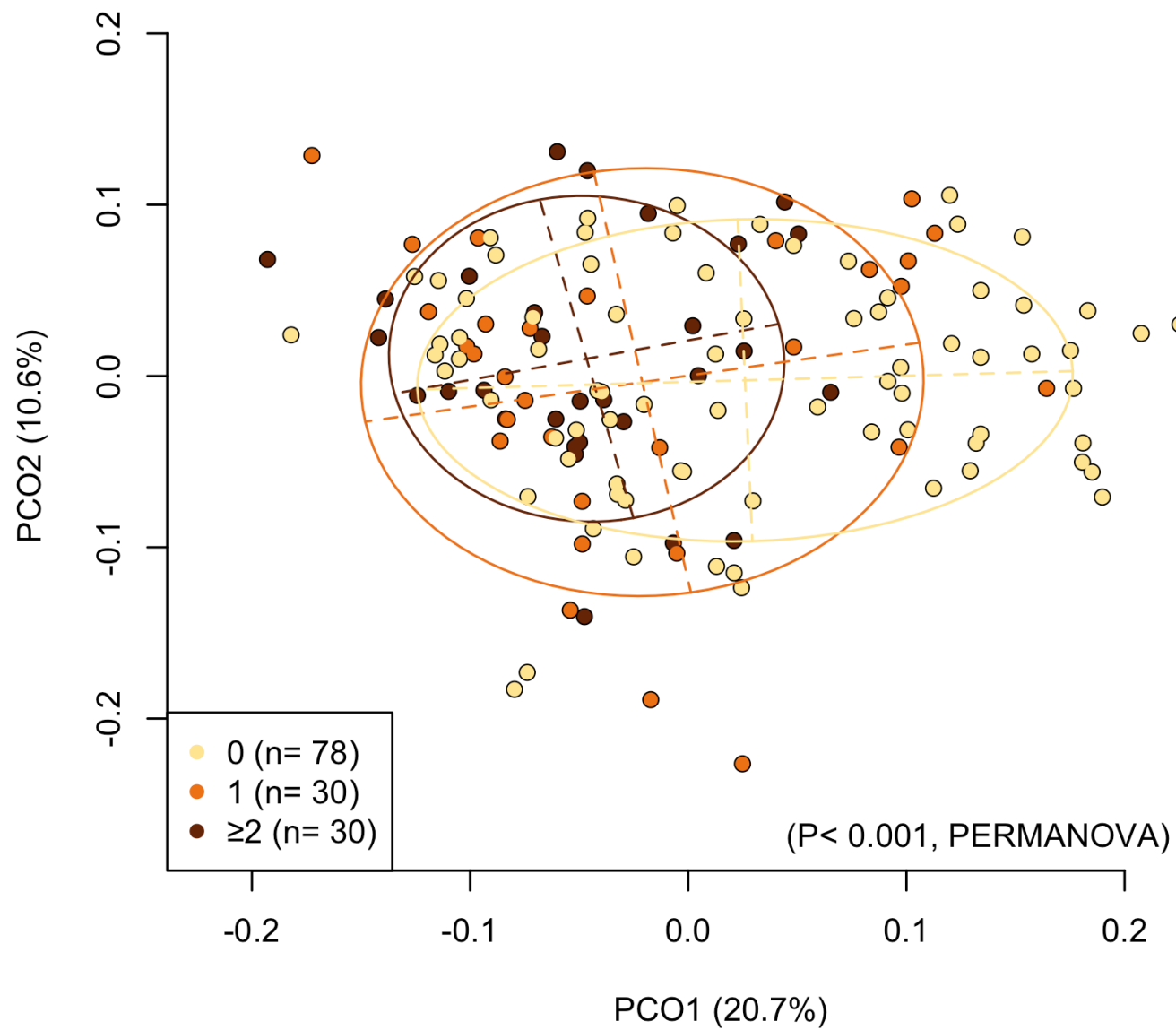

Supplement: FIG S4 [file mSphere.01252-20-sf004.pdf]

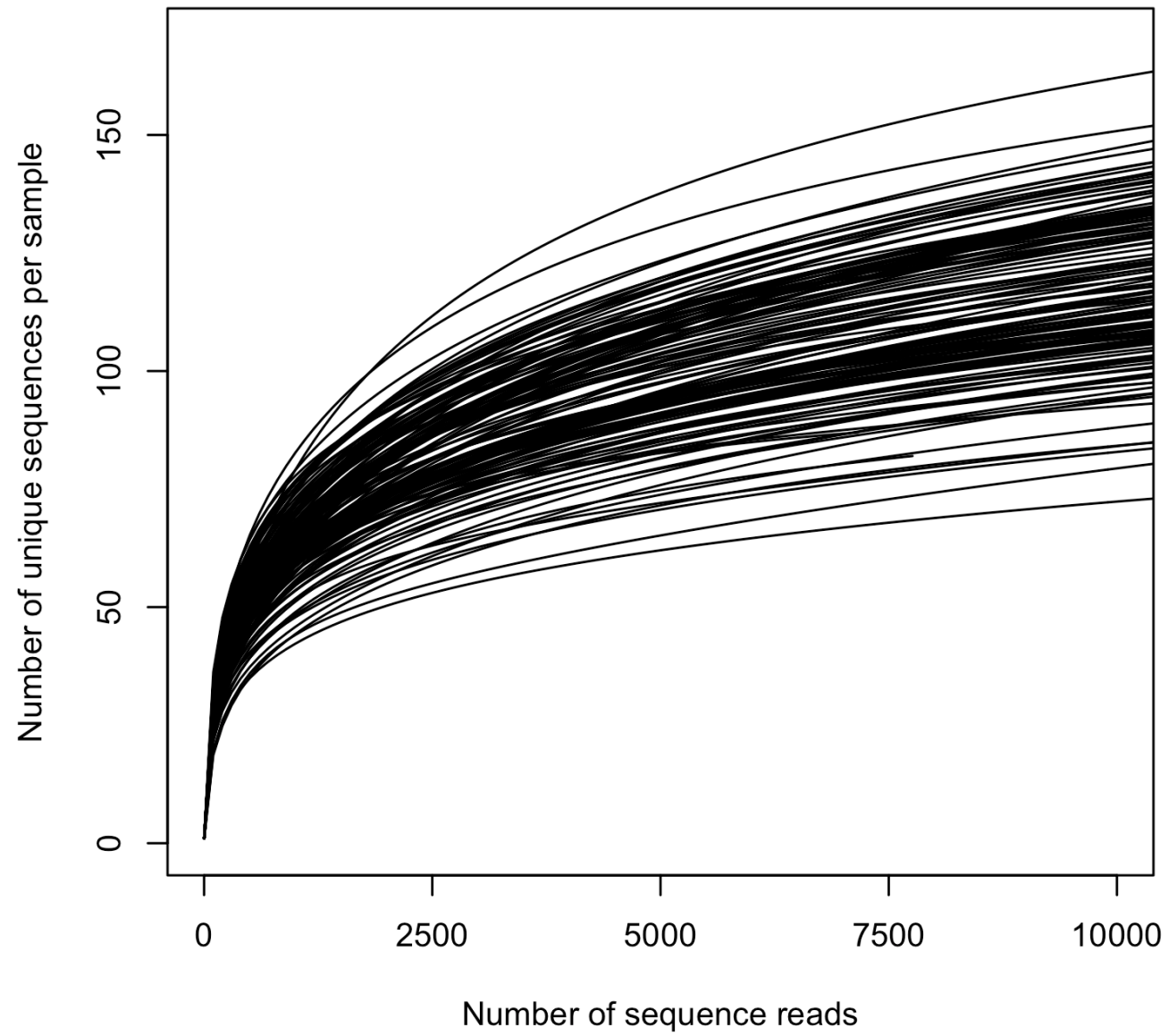

Supplement: FIG S5 [file mSphere.01252-20-sf005.pdf]
