# Supplementary material for: Tongue Microbiota Composition and Dental Caries Experience in Primary School Children
Source: mSphere. 2021 Apr 28;6(2):e01252-20. doi: 10.1128/mSphere.01252-20 (PMC8092142; doi:10.1128/mSphere.01252-20)
Supplement: TABLE S1 [file mSphere.01252-20-st001.docx]

Table S1. Bacterial taxa corresponding to OTUs belonging to commensal groups I and II.

| OTU  no. | Bacterial taxa  corresponding to each OTU | Relative abundance (%) | No. ^a^ |
| --- | --- | --- | --- |
| Commensal group I | |  |  |
| OTU3 | *Prevotella melaninogenica* (469) | 12.0±7.3 |  |
| OTU7 | *Streptococcus salivarius* (755) | 7.4±6 |  |
| OTU10 | *Veillonella parvula* (161) | 6.8±3.6 |  |
| OTU5 | *Prevotella histicola* (298) | 3.5±4.5 |  |
| OTU13 | *Prevotella pallens* (714) | 1.6±1.3 |  |
| OTU15 | *Leptotrichia* sp. (417) | 1.1±2.3 |  |
| OTU12 | *Solobacterium moorei* (678) | 1.1±0.9 |  |
| OTU133 | *Prevotella* sp. (313) | 1.0±1.7 |  |
| OTU38 | *Streptococcus parasanguinis* (411) | 1.0±1.1 |  |
| OTU17 | *Campylobacter concisus* (575) | 0.8±0.6 |  |
| OTU20 | *Prevotella* sp. (306) | 0.6±1.5 |  |
| OTU26 | *Lachnospiraceae* bacterium (096) | 0.5±1.3 |  |
| OTU32 | *Alloprevotella rava* (302) | 0.4±1.0 | 5 |
| OTU42 | *Prevotella salivae* (307) | 0.4±0.6 | 7 |
| OTU18 | *Atopobium parvulum* (723) | 0.4±0.6 | 1 |
| OTU24 | *Peptostreptococcaceae* *sulci* (467) | 0.3±0.3 | 3 |
| OTU41 | *Megasphaera micronuciformis* (122) | 0.2±0.3 | 6 |
| OTU241 | Genus *Actinomyces*^b^ | 0.1±0.2 | 4 |
| OTU96 | *Streptococcus parasanguinis* (411) | 0.1±0.1 | 10 |
| OTU67 | *Veillonella atypica* (524) | 0.1±0.2 | 8 |
| OTU73 | *Prevotella* sp. (305) | 0.1±0.1 | 9 |
| OTU186 | *Streptococcus salivarius* (755) | 0.1±0.1 | 2 |
| Commensal group II | |  |  |
| OTU4 | *Neisseria subflava* (476) | 12.1±9.2 |  |
| OTU1 | *Fusobacterium periodonticum* (201) | 4.5±3.5 |  |
| OTU6 | *Haemophilus parainfluenzae* (718) | 4.4±4.5 |  |
| OTU8 | *Porphyromonas pasteri* (279) | 3.8±4.0 |  |
| OTU34 | *Streptococcus oralis* ss. *dentisani* (058) | 1.0±1.2 |  |
| OTU16 | *Gemella sanguinis* (757) | 1.0±0.8 |  |
| OTU14 | *Porphyromonas* sp. (284) | 0.9±1.7 |  |

^a^Numbers used for indicating the OTUs in Figure 1. ^b^No blast hit with ≥98.5% identity was found in the expanded Human Oral Microbiome database (eHOMD). Oral taxon IDs in eHOMD are given in parentheses following bacterial names.
